# Supplementary material for: Unfolding of α-helical 20-residue poly-glutamic acid analyzed by multiple runs of canonical molecular dynamics simulations
Source: PeerJ. 2018 May 15;6:e4769. doi: 10.7717/peerj.4769 (PMC5958886; doi:10.7717/peerj.4769)
Supplement: Supplemental Information 1 [file peerj-06-4769-s001.docx]

Table S1. Probabilities of secondary structure transitions, $P^{Sim}(y,x;i)$.

| *i* | 2 | | | |  | 11 | | | |  | 19 | | | |
| --- | --- | --- | --- | --- | --- | --- | --- | --- | --- | --- | --- | --- | --- | --- |
| *x* \ *y* | $H$ | $T$ | $G$ | $\bar{HTG}$ |  | $H$ | $T$ | $G$ | $\bar{HTG}$ |  | $H$ | $T$ | $G$ | $\bar{HTG}$ |
| $H$ | 0.91 | 0.04 | 0.03 | 0.02 |  | 0.95 | 0.04 | 0.01 | 0.00 |  | 0.56 | 0.35 | 0.01 | 0.08 |
| $T$ | 0.03 | 0.78 | 0.03 | 0.15 |  | 0.10 | 0.72 | 0.07 | 0.11 |  | 0.20 | 0.60 | 0.04 | 0.17 |
| $G$ | 0.15 | 0.17 | 0.56 | 0.10 |  | 0.08 | 0.30 | 0.51 | 0.02 |  | 0.05 | 0.30 | 0.49 | 0.14 |
| $\bar{HTG}$ | 0.00 | 0.03 | 0.00 | 0.97 |  | 0.00 | 0.03 | 0.01 | 0.96 |  | 0.01 | 0.02 | 0.00 | 0.97 |
