# Supplementary material for: Unfolding of α-helical 20-residue poly-glutamic acid analyzed by multiple runs of canonical molecular dynamics simulations
Source: PeerJ. 2018 May 15;6:e4769. doi: 10.7717/peerj.4769 (PMC5958886; doi:10.7717/peerj.4769)
Supplement: Supplemental Information 2 [file peerj-06-4769-s002.docx]

Table S2. Probabilities of helix folding and unfolding in *Sim* runs.

|  | All | *N*^*1^ | *M*^*2^ | *C*^*3^ |
| --- | --- | --- | --- | --- |
| $P^{Sim}(H, HHH)$ | 0.96 | 0.97 | 0.98 | 0.92 |
| $P^{Sim}(-, HH-)$ | 0.29 | 0.31 | 0.24 | 0.30 |
| $P^{Sim}(-,-HH)$ | 0.09 | 0.07 | 0.09 | 0.30 |
| $P^{Sim}(H, H--)$ | 0.26 | 0.15 | 0.24 | 0.29 |
| $P^{Sim}(H, --H)$ | 0.04 | 0.03 | 0.04 | 0.05 |
| $P^{Sim}(H, H-H)$ | 0.07 | 0.02 | 0.11 | 0.06 |
| $P^{Sim}(H, ---)$ | 0.02 | 0.02 | 0.01 | 0.02 |

^*1^ The N-terminal region consisting of the 2nd–7th residues. ^*2^ The middle region consisting of the 8th–13th residues. ^*3^ The C-terminal region consisting of the 14th–19th residues.
