# Supplementary figures and images for: Unfolding of α-helical 20-residue poly-glutamic acid analyzed by multiple runs of canonical molecular dynamics simulations
Source: PeerJ. 2018 May 15;6:e4769. doi: 10.7717/peerj.4769 (PMC5958886; doi:10.7717/peerj.4769)

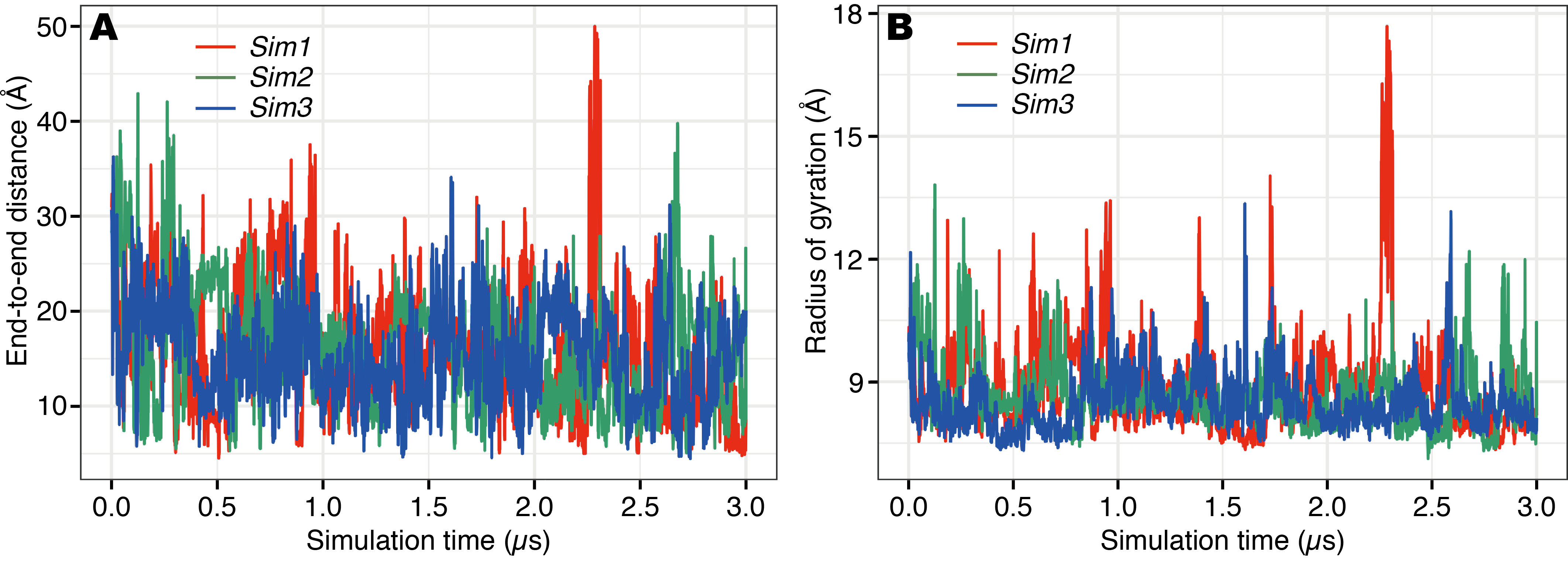

Supplement: Supplemental Information 3 [file peerj-06-4769-s003.png]

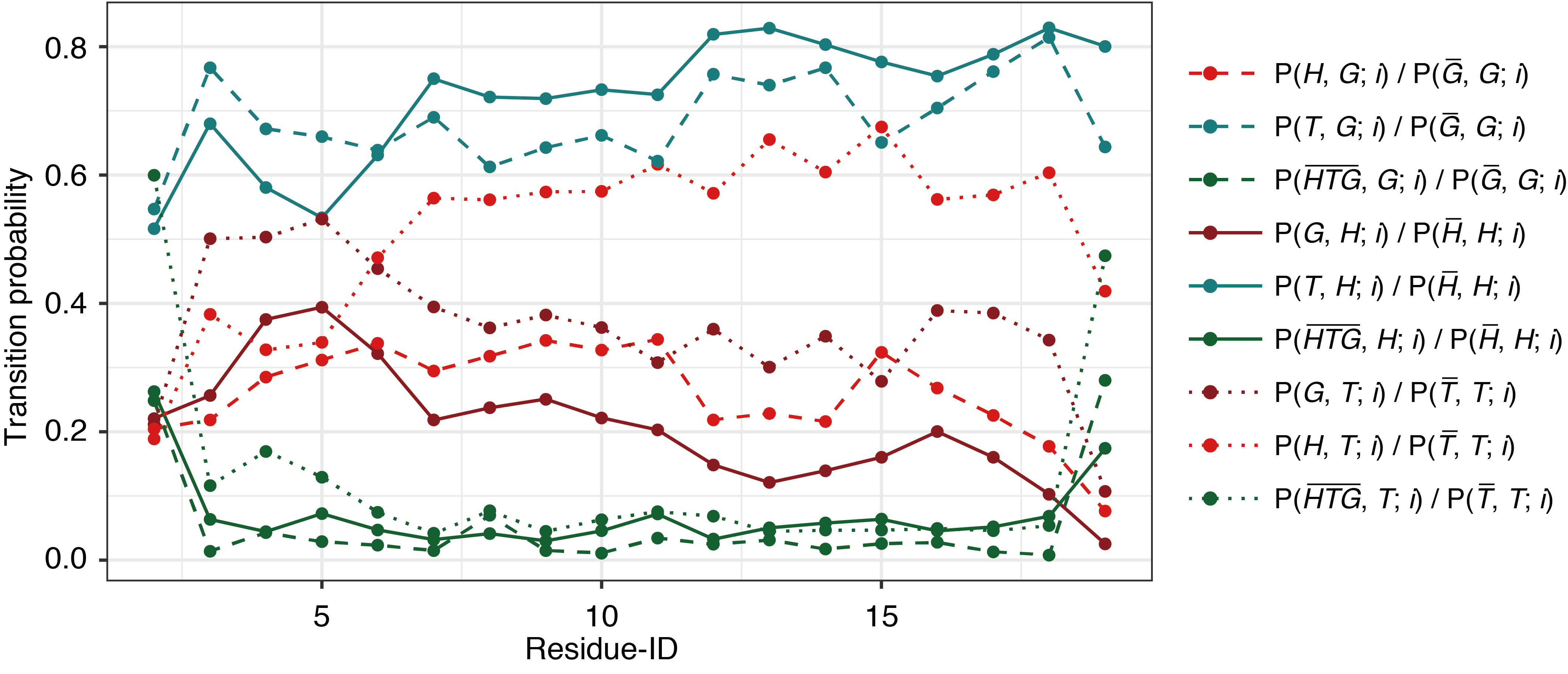

Supplement: Supplemental Information 4 — The horizontal axis indicates the position of each residue, i. Transitions from α-helix (H), 310-helix (G), and turn (T) are shown in solid, dashed, and dotted lines, respectively. Destination states are indicated as red, maroon, dark-cyan, and green, for H, G, T, and structural elements other than these, respectively. [file peerj-06-4769-s004.png]
